# Physical performance and compensation strategies of older adults to maintain physical fitness and well-being during the COVID-19 pandemic in Germany

**DOI:** 10.1186/s12877-023-03952-9

**Published:** 2023-04-20

**Authors:** Torben Gehle, Sandra Lau, Michel Hackbarth, Tania Zieschang, Jessica Koschate

**Affiliations:** School of Medicine and Health Sciences, Department for Health Services Research, Geriatric Medicine, Carl von Ossietzky University Oldenburg, Oldenburg, Germany

**Keywords:** COVID-19 pandemic, Physical training habits, Subjective well-being, Exercise training, Chip-controlled fitness circuit

## Abstract

**Introduction:**

During the first wave of the COVID-19 pandemic in March 2020, worldwide restrictions in social life, including the closure of sport facilities, led to a reduction of physical activity and subjective well-being. The aim of this study is to describe physical training habits, and subjective well-being in relation to objective training data from a chip-controlled fitness circuit in the rural area of Oldenburg, Germany.

**Materials and methods:**

Overall, 35 older adults (20 women 71 ± 6 y/o and 15 men, 72 ± 7 y/o), regularly exercising in a chip-controlled fitness circuit before the lockdown in March 2020, were interviewed. The training data from February to August 2020 from six strength and two endurance exercise devices were extracted and compared to data before and up to three months after the lockdown. Additionally, participants’ personal characteristics, physical activities and quality of life before, during, and after the first lockdown were assessed.

**Results:**

The leg score (pre, post_June_, post_July_, post_August_: 1207 ± 469 kg, 1248 ± 477 kg, 1254 ± 516 kg, 1283 ± 493 kg; p = 0.137) and endurance scores (ergometer: 0.93 ± 0.35 min^− 1^ · watt^− 1^, 0.86 ± 0.31 min^− 1^ · watt^− 1^, 0.86 ± 0.31 min^− 1^ · watt^− 1^, 0.85 ± 0.28 min^− 1^ · watt^− 1^ ; p = 0.442) were not significantly different, in contrast to the rowing score (1426 ± 582 kg, 1558 ± 704 kg, 1630 ± 757 kg, 1680 ± 837 kg; p < 0.001). A significant increase of total energy expenditure (p = 0.026), mainly through gardening, walking, and bike riding was observed. The greatest personal limitation reported, was the loss of social contacts.

**Conclusion:**

The presented data did not show a decrease in training performance, but a slight trend towards an increase. A compensatory increase in regular outdoor activities seems to have a protective effect against a loss of training performance and may have the potential to stabilize subjective well-being during lockdown periods.

## Introduction

In the context of the first wave of the COVID-19 pandemic, not only in Germany, but global strict measures to control the pandemic were introduced in March 2020. Besides retail shops and cultural facilities, all institutions for structured sports activities in Germany were closed.

During these so-called lockdown periods, reduced physical activity, as a consequence of the restrictions, were reported in several countries [11]. Especially the previously very active, well-trained individuals showed greater decline in physical activity, compared to formerly moderately active individuals, or those with a low level of physical activity [10,12]. The analysis of a large anonymized data set of 17,450 participants, training in a chip-controlled fitness circuit, supported the findings of reduced physical activity by showing that training performance after the pandemic lockdown decreased substantially in the individuals training with the highest intensity prior to the lockdown [12].

Consequences of reduced physical activity, as shown in detraining-studies, are a decrease in subjective well-being [2,3,10], significant losses in muscle mass and strength as well as metabolic changes in older individuals, like reduced postprandial insulin sensitivity, synthesis of muscle protein and increased TNF-α and CRP [6–9, 15]. Previous training studies in older people showed that physical activity is very important for quality of life [1–3], prevention of sarcopenia [4] and thus falls, which may lead to a need for long-term care [5].

The analysis of the large anonymous data set of the CoNFINE study [12] showed the effect of the pandemic-related gym closure on objective exercise data, however, no further information on health status, physical activity habits or compensation strategies during the lockdown period were available.

The aim of this study is to describe physical activity habits, health conditions and subjective well-being of older individuals in Oldenburg and its rural surrounding area, in a small subgroup of the data published by Zieschang et al. [12], during the first lockdown period of the COVID-19 pandemic in Germany. Moreover, the effects of the respective level of physical activity during the lockdown on the training data obtained from the fitness circuit after the lockdown will be presented. We expect a decrease in physical activity and subjective well-being.

## Methods

### Study design

In this observational study, the recorded training data from a chip-controlled fitness circuit (milon industries, Emersacker, Germany), which includes strength and endurance exercise devices, were retrospectively extracted for the time intervals 4 weeks before the lockdown, and up to 3 months after the lockdown of the first wave of the COVID-19 pandemic. Data were extracted, according to the General Data Protection Regulation of the European Union. Furthermore, participants were interviewed via telephone, using questionnaires at three different time points. Prior to the beginning of the study, a positive vote of the medical ethical committee of the Carl von Ossietzky University Oldenburg was available (No. 2020-061), and the study was registered at the German clinical trials registry (DRKS00022433).

### Participants

The participants were recruited in cooperation with three physiotherapy practices or gyms in the rural area of Oldenburg, offering exercise training in a specific chip-controlled fitness circuit (milon®), which includes strength and endurance exercise training devices. The owners of the physiotherapy practices were contacted via telephone by the study personnel and asked to reach out to their clients to inform them about the study via phone. Owners and participants were offered a financial compensation for their participation. In the event that the clients were interested in participating in the study, they received the contact data of the study team. Informed consent was then obtained via telephone, or, if requested by the participant, via (E-) mail, prior to the beginning of the interview. Participants of the study had to be at least 60 years of age, and had to be regularly exercising in the milon® strength and endurance exercise circuit before the lockdown in March 2020. Participants, with no registered training during the month prior to the lockdown were excluded from the study. The data were pseudonymized by assigning a six-digit ID to each participant.

### Telephone interviews

**Fig. 1:**
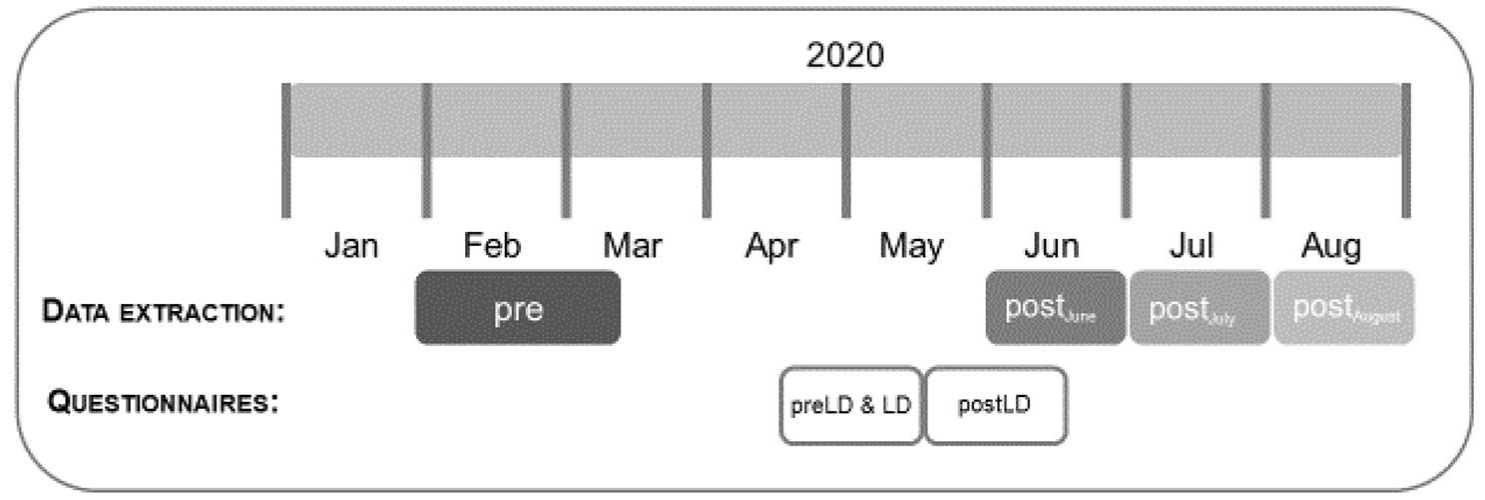
Description of the schedule for the interviews and the data extraction from the milon cloud *Time periods: pre: February to March 2020; post*_*June*_: *June 2020; post*_*July*_: *July 2020; post*_*August*_: *August 2020. preLD: before the lockdown, LD: during the lockdown, postLD: after the lockdown.*

The semi-structured telephone interviews were conducted through various experimenters by the study team during two time periods. The first interviews were conducted between April 23^rd^ and May 20^th^ in 2020. Two questionnaires were filled in at this time point, assessing the individual situation before (preLD) and during the lockdown (LD). The second interval for interviews was from May 19^th^ until June 30 ^st^ in 2020, which was directly after the reopening of sports facilities after the first lockdown (postLD) (Fig. 1). Ahead of all interviews, the study team was trained to perform the interviews in a standardized manner.

The semi-structured interviews included personal characteristics, as age, gender, marital status, living situation, education and occupation, pre-existing medical diagnoses, subjective pain during rest, as well as standardized questionnaires, as the Minnesota leisure time physical activity questionnaire, the short Falls Efficacy Scale International (short FES-I) assessing concerns of falling in certain situations in 7 questions with a 4 items Likert scale [17] (higher values indicate greater fear of falling), the health status questionnaire (SF-12) [18], a shortened version of the SF-36, which can be used to assess subjective physical and psychological quality of life, using 12 questions with 2 to 6 item Likert scales. Additionally, the LUCAS (Longitudinal Urban Cohort Ageing Study) index [16] was assessed, which evaluates functional competence based on 12 questions for people above the age of 60 years with no previous need for long-term care, and allows an assessment of the risk of needing long-term care. The LUCAS-scores were used to categorize participants as either frail, pre-frail, post-robust or robust. The data from before the lockdown were assessed retrospectively during the first interview, with a moderate delay of 6 weeks.

### Database of the chip-controlled fitness circuit

The fitness circuit includes 6 resistance training devices (abdominal curl, back extension, chest press, leg curl, leg extension, rowing) and 2 endurance training devices (cross trainer, cycle ergometer). After the reopening of the gyms, training was possible under certain conditions, such as a maximum number of visitors, keeping a minimum distance between visitors, wearing masks except on the equipment and disinfection before and after using the equipment.

For the strength training, the number of training sets (sets), the average weight of the concentric load, while lifting the weight to the final position (avg weight), the average weight of the eccentric load while releasing the weight back to the starting position (avg delta weight), the average work on the device (worksum), the overall number of repetitions on the device (moves), the average number of repetitions for all sets of the session on the device (avg moves), the maximum heart rate (HR_max_) and the average heart rate (HR_mean_) in case the participants wore the chest strap, and the time spent on the device (duration) were recorded.

For the endurance training devices, the number of training sessions (sets), the average work (worksum), revolutions per minute (avg rpm), HR_max_ and HR_mean_ (in case the participants wore the chest strap), average power (avg watt), time spent on the device (duration) and the number of repetitions performed on the device (sets) were recorded. According to the intended training protocol of the circuit, the resistive exercise devices were used for 60 s each, and the endurance devices for 240 s each, with 30 s break in-between to switch between the devices.

Data of the participants were exported pseudonymously from the milon® care cloud, by the data scientist of milon industries. Milon industries received no information about the telephone interviews and a second 6-digit pseudonym was created for the data export from the milon care cloud. Only the study team was able to match the data from the milon care cloud with the data obtained during the telephone interviews. All training data during the period from February 1^st^, until August 31^st^ 2020 were exported.

### Data analyses

Four training time points for the data of the chip-controlled fitness circuit were set from February to March (pre) and the three months after the lockdown June (post_June_), July (post_July_) and August (post_August_) (Fig. 1). Following the previous studies, the leg score as the primary outcome (product of training weight on the leg extensor (sum of lifting and lowering the weight) and the overall number of repetitions) was calculated. All other calculated parameters were defined as secondary outcomes, such as the rowing score (product of training weight on the rowing machine (sum of lifting and lowering the weight) and the overall number of repetitions) and the endurance score (quotient of HR and average watt) [12]. For each time point, mean values as further secondary outcomes were calculated for all parameters of each training device and compared over the four time points. In addition, the individual training break was calculated as the days between the last training before and the first training after the lockdown.

Furthermore, the subgroup of participants with a high exercise level was examined, which was defined according to the group with the most intense training regime (baseline leg score > 1320 kg) in the data of Zieschang et al. [12]. Leg, rowing, and endurance scores were compared over the four time points for this group.

In addition, the age-specific target heart rate was calculated for each participant (220-age) and was then compared to the average heart rate during training to evaluate the respective training intensity.

For preparation for the statistical analysis, data of the milon circuit were imported to Microsoft Access 2019 version 1808.

Initially, the interviews were documented on paper and the results of the analysis were defined as secondary outcomes. Afterwards, two independent members of the study team entered the data into the REDCap data management system [21,22], and a third person performed a review to identify and correct any inconsistencies, based on the original interview files.

The activities in minutes, reported for the Minnesota leisure time physical activity questionnaire were converted to energy expenditure in kilocalories for further analyses, using the values provided by the Compendium of Physical Activities [19].

The total energy expenditure including all activities was calculated and for further analyses, only activities that were not affected by the restrictions during the lockdown and undertaken by at least 5 individuals at each of the three time points were considered, to ensure a sufficiently high number of data points for the analysis. These included the following activities: walking, lawn mowing, gardening, bicycling and jogging.

The physical and the psychological sum scales were calculated according to the SPSS-based algorithms, provided within the manual for the SF-12 [18].

### Statistical analyses

Statistical analyses were performed, using SPSS 26 (IBM, Amonk, News York, USA). If normal distribution of the data could be assumed (α ≤ 10%), ANOVA with repeated measures on the factor training session (pre, post_June_, post_July_, post_August_) was used to calculate differences over time for the eight training devices of the milon circuit, respectively. Bonferroni post hoc tests were used to compare the sessions individually. If sphericity could not be assumed, the main effects were corrected according to Huynh-Feldt. In case normal distribution could not be assumed, a non-parametric test according to Friedman with Wilcoxon post-hoc tests, applying the Bonferroni correction, were performed. The evaluation of the questionnaires for the three time points (preLD, LD, post LD) was performed similarly, as well as for the results of the LUCAS-Index, the SF-12 and the Minnesota leisure time physical activity questionnaire.

For explorative correlation analyses at the three time points preLD, LD and postLD between the performed specific activities in daily life and total energy expenditure, Pearson tests were used, if normal distribution of the data could be assumed. In case normal distribution could not be assumed, Spearman tests were applied. The results of the correlation analyses were classified into weak (r < 0.400), moderate (r ≥ 0.400 < 0.600) and strong (r ≥ 0.600) [24]. The significance level was set to α = 5%.

## Results

With the help of the physiotherapy practices, a total of 35 volunteers were acquired. All 35 participants answered all three questionnaires completely. After receiving the milon data, it was apparent that one participant had not exercised prior to the lockdown and was therefore not included in the calculations of performance in the fitness circuit. Anthropometric data of the participants are shown in Table 1.

**Table 1:** Participants’ characteristics

	n	Age [years] mean ± SD (min-max)	Retired [n]	height [cm] mean ± SD (min-max)	body mass [kg] mean ± SD (min-max)	BMI [kg·m^−^²] mean ± SD (min-max)
**overall**	35	71 ± 6 (61–85)	30	172 ± 10 (150–193)	79 ± 14 (58–114)	26.5 ± 3.8 (18.8–36)
**women**	20	71 ± 6 (61–82)	16	166 ± 7 (150–177)	73 ± 13 (58–97)	26.4 ± 4 (19.7–35)
**men**	15	72 ± 7 (61–85)	14	181 ± 7 (170–193)	87 ± 13 (70–114)	26.6 ± 3.7 (18.8–36)
**Intense exercise group (leg score > 1320 kg)**						
**overall**	13	70 ± 7 (61–85)	10	175 ± 9 (162–190)	80 ± 14 (60–104)	26.±2.7 (22–30)
**women**	5	69 ± 5 (62–73)	3	167 ± 6 (162–177)	67 ± 7 (60–78)	24.1 ± 2.7 (22–28,7)
**men**	8	71 ± 8 (61–85)	7	179 ± 7 (170–190)	88 (71–104)	27.2 ± 2.1 (24.6–30)

SD: standard deviation; BMI: body mass index

### Interview data

During the first interview, of the 35 participants, 28 persons reported to be married or to have a life partner, and all but 5 participants had one or more children. 28 lived with their partner and 3 received further help (home help or care service), 7 lived alone with one participant receiving help (help for housekeeping or care service). 30 participants lived in a single-family house, the other 5 in apartments. The majority (n = 20) had completed an apprenticeship or graduated from university (n = 12).

Neither the family situation, nor the occupational or the housing situation had changed over time. Pre-existing conditions, the participants reported, were mainly orthopaedic diseases (n = 24) such as osteoarthritis of the large and small joints such as the spine, hip, shoulder, knee and fingers, cardiovascular diseases (n = 21) such as arterial hypertension, coronary heart disease or atrial fibrillation and neurological diseases (n = 9) such as polyneuropathy. Two participants had a level of care (level 1 and level 2). No onset of new diseases was reported during the study period, including, fortunately, no infection with SARS CoV 2.

The personal restrictions included a mask requirement, regular disinfection and contact restrictions with people outside the household. In addition, private international travel was prohibited and, if possible, work was to be done in a home office. All leisure activities or visiting care facilities (cinema, gym, concerts, meetings, religious gatherings, schools, kindergartens, visits to nursing homes or hospitals, etc.) were not possible, due to government regulations. The individual definition for the onset of personal restrictions in the context of the lockdown period of the first COVID-19 wave in Germany was reported as follows: seven participants reported it as the call to restrict social contacts on February 12^th^ 2020, 12 mentioned the closing of stores on March 17^th^ 2020, and 14 the restriction to not meet more than 2 people at the same time on March 22^nd^ 2020. The most burdensome restriction during the pandemic for most participants was the restriction of social contacts (n = 25), which was followed by the burden of the restrictions in public life (n = 3) and the restriction of sports activities (n = 3). One participant stated that the fear of the infection with Sars-Cov2 was the most limiting factor, no participant stated financial worries as a burden.

**Fig. 2:**
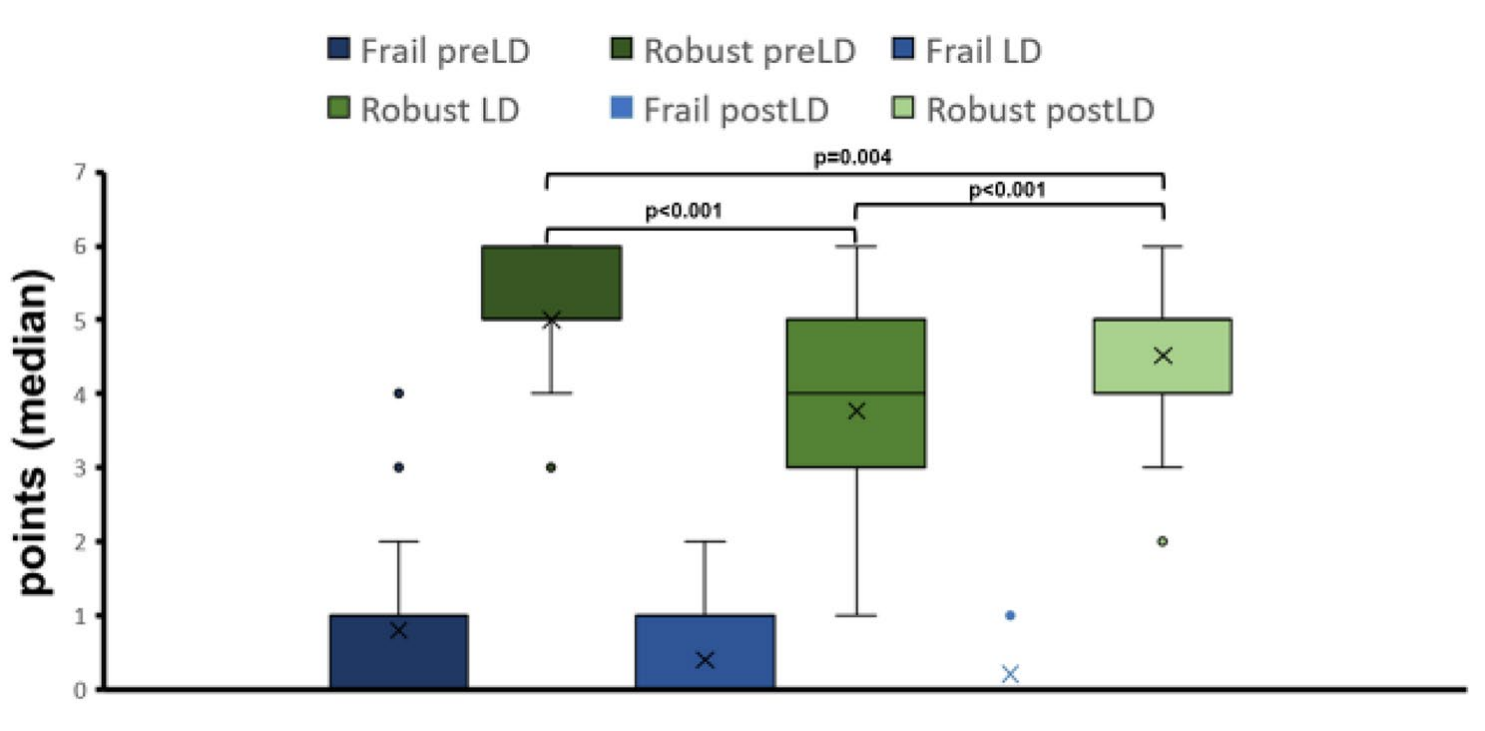
Median for the LUCAS functional index for Frail and Robust *preLD: before the lockdown, LD: during the lockdown, postLD: after the lockdown*

In the standardized questionnaires, a trend was demonstrated in the SF-12 in the psychological sum scale overall (preLD: 56 ± 6, LD: 55 ± 6, postLD: 56 ± 7, p = 0.053). No significant difference was observed in the physical sum scale (preLD: 46 ± 9, LD: 46 ± 10, postLD: 46 ± 8, p = 0.584)), or in the Short-FESI (preLD: 8.4 ± 2, LD: 8.4 ± 2, postLD: 8.0 ± 1, p = 0.943) and the LUCAS functional index for the FRAIL score (median = 0/0/0, p = 0.083). In the LUCAS functional index for ROBUST, a highly significant difference was demonstrated over time (p < 0,001), as well as between the different timepoints (post hoc results shown in Fig. 2).

Energy expenditure was significantly different. Especially energy expenditures for activities as walking, gardening and riding the bike increased significantly over time (Table 2).

**Table 2:** Energy expenditure via daily activities over a period of the past two weeks converted into kcal

			total energy expenditure per activity [kcal]			Statistical analyses				
activity	participants	possible during LD	preLD	LD	postLD	overall	preLD to LD	LD to postLD	preLD to postLD	
gym	35	no								
rehab sports	6	no								
physiotherapy	2	no								
milon circuit	35	no	531	0	393	p < 0.001	p < 0.001	p < 0.001	p < 0.001	
walking	26	yes	473	650	519	p = 0.067	p = 0.018	p = 0.139	p = 0.693	
lown mowing	9	yes	113	123	69	p = 0.045	p = 0.317	p = 0.043	p = 0.08	
gardening	27	yes	914	1236	1486	p = 0.047	p = 0.021	p = 0.839	p = 0.074	
hiking	1	yes	82	82	113	p = 0.368				
bike riding	24	yes	517	880	1176	p = 0.009	p = 0.010	p = 0.113	p = 0.001	
bicycle ergometer (home)	3	yes	46	50	46	p = 0.949				
dancing	0	no	0	0	0					
jogging/ walking	5	yes	79	70	77	p = 0.124				
swimming	5	no	62	0	60	p = 0.066			p = 0.865	
sailing	0	yes	0	0	0					
tennis	0	no	0	0	0					
badminton	0	no	0	0	0					
golf	2	no	137	0	111	p = 0.135			p = 0.317	
kcal in total*			3117	3254	4174	p = 0.026	p = 0.713	p = 0.025	p = 0.004	

*including all activities of the questionnaire

*preLD: before the lockdown, LD: during the lockdown, postLD: after the lockdown*

### Training data

The objective training data were extracted and analysed as in the large data set of the CoNFINE study [12].

The individual training break was 82 days (± 14 days).

**Fig. 3:**
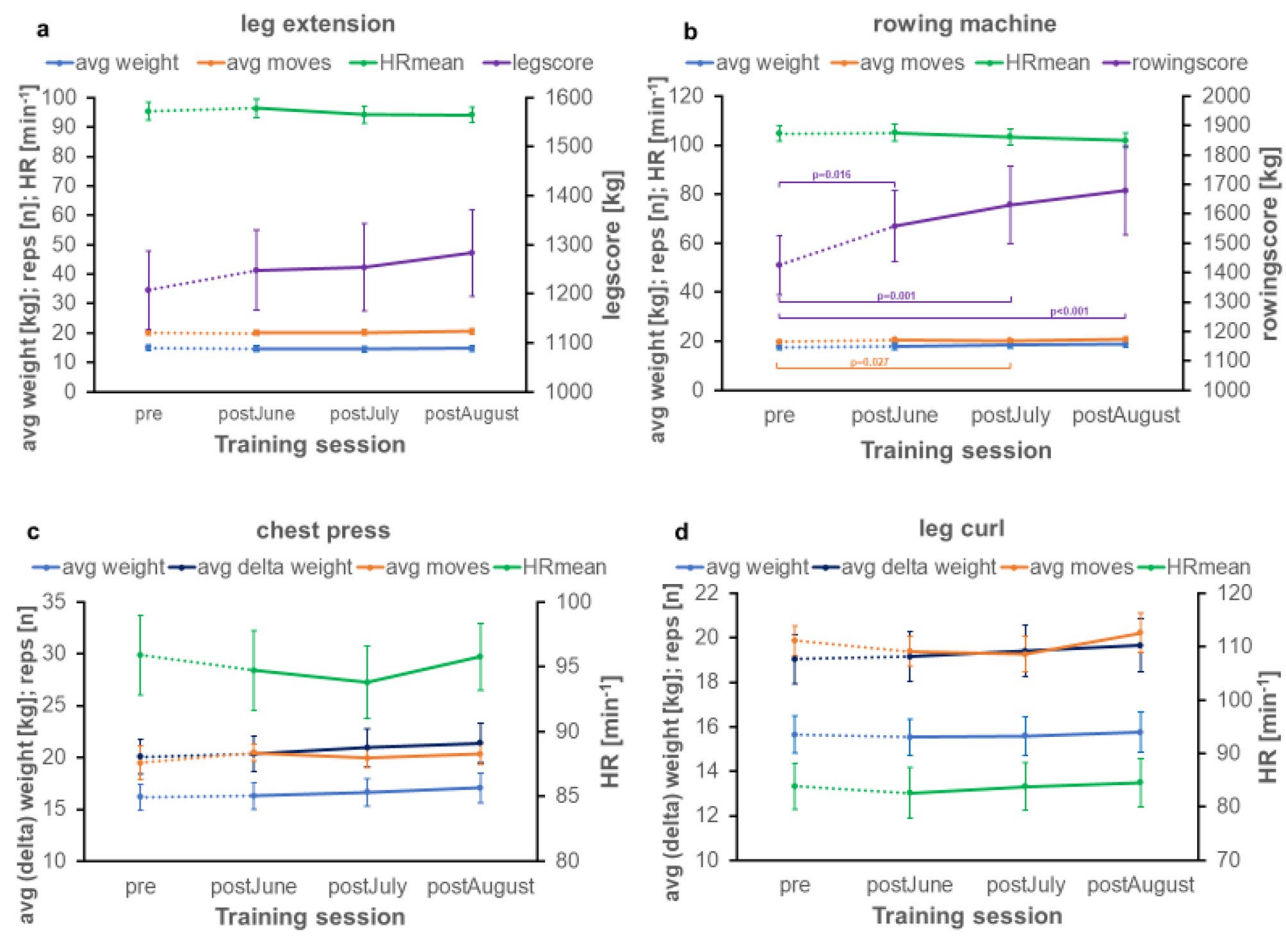
Training data exported from the resistance exercise devices *Time periods: pre: February to March 2020; post*_*June*_: *June 2020; post*_*July*_: *July 2020; post*_*August*_: *August 2020. avg weight: mean weight by lifting the weight to the final position; avg moves: mean number of repetitions; HR*_*mean*_: *mean heartrate at the device; avg delta weight: mean weight by releasing the weight to the starting position. 25–28 participants have worn the belt. Panel a: average weight, average moves, average HR and leg score on the leg extension device from February to August 2020. Panel b: average weight, average moves, average HR and rowingscore on the rowing machine from February to August 2020. Panel c: average weight, average delta weight, average moves and average HR on the chest press device from February to August 2020. Panel d: average weight, average delta weight, average moves and average HR on the leg curl device from February to August 2020.*

**Fig. 4:**
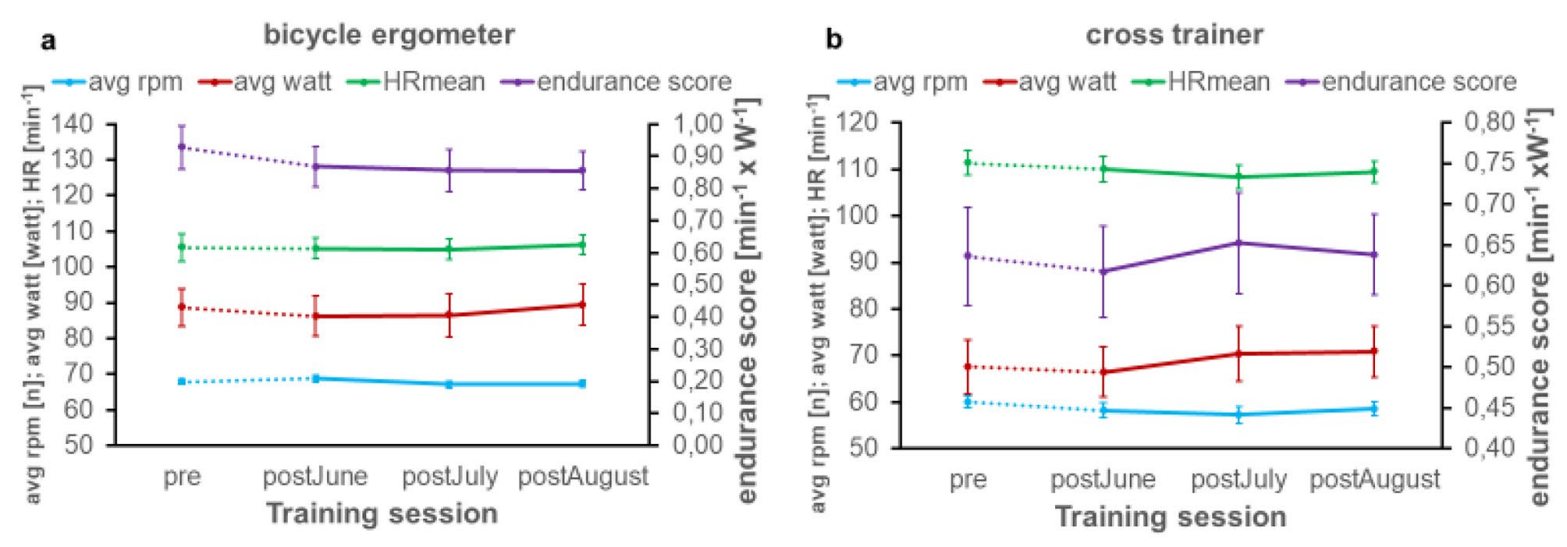
Training data exported from the endurance exercise devices *Time periods: pre: February to March 2020; post*_*June*_: *June 2020; post*_*July*_: *July 2020; post*_*August*_: *August 2020. avg rpm: mean revolutions per minute on the device; avg watt: mean power in watt on the device; HR*_*mean*_: *mean heart rate on the device. Panel a: average rounds per minute, average watt, average HR and endurance score on the bicycle ergometer from February to August 2020. Panel b: average rounds per minute, average watt, average HR and endurance score on the cross trainer device from February to August 2020.*

The mean leg score before the lockdown was 1207 kg. The leg score showed no significant differences over time (p = 0.137) (Fig. 3a). The rowing score increased over time (p < 0.001) with significant differences between pre and all other three time points (Fig. 3b). The bicycle ergometer endurance score (p = 0.442) showed no significant differences as well as for the crosstrainer endurance score (p = 0.092) (Fig. 4a + b).

In summary, the analysis of the detailed parameters of the training devices, as avg weight, avg delta weight, worksum, moves, avg moves, HR_max_, HR_mean_, avg rpm, avg watt, showed barely any significant differences and only a few trends:

For the seated row device, significant effects for time were observed in delta avg weight (p = 0.025, η²=0.139), worksum (p < 0.001), moves (p = 0.003 η²=0.167) and avg moves (p = 0.024 η²=0.134) with some significant differences in the post-hoc analysis (Fig. 3b), as well as a trend for avg weight (p = 0.055, η²=0.101).

For the chest press device, in the main effect a trend was seen between the four time points in avg moves (p = 0.055; η²=0.099).

The group was divided in thirds according to their training intensity before the lockdown. In this small sample, 13 participants belonged to the high training intensity group (mean leg score = 1696 kg ± 246 kg), 12 to the medium group (mean leg score = 1114 kg ± 122 kg) and 9 to the low intensity group (mean leg score = 626 kg ± 123 kg), respectively.

**Fig. 5:**
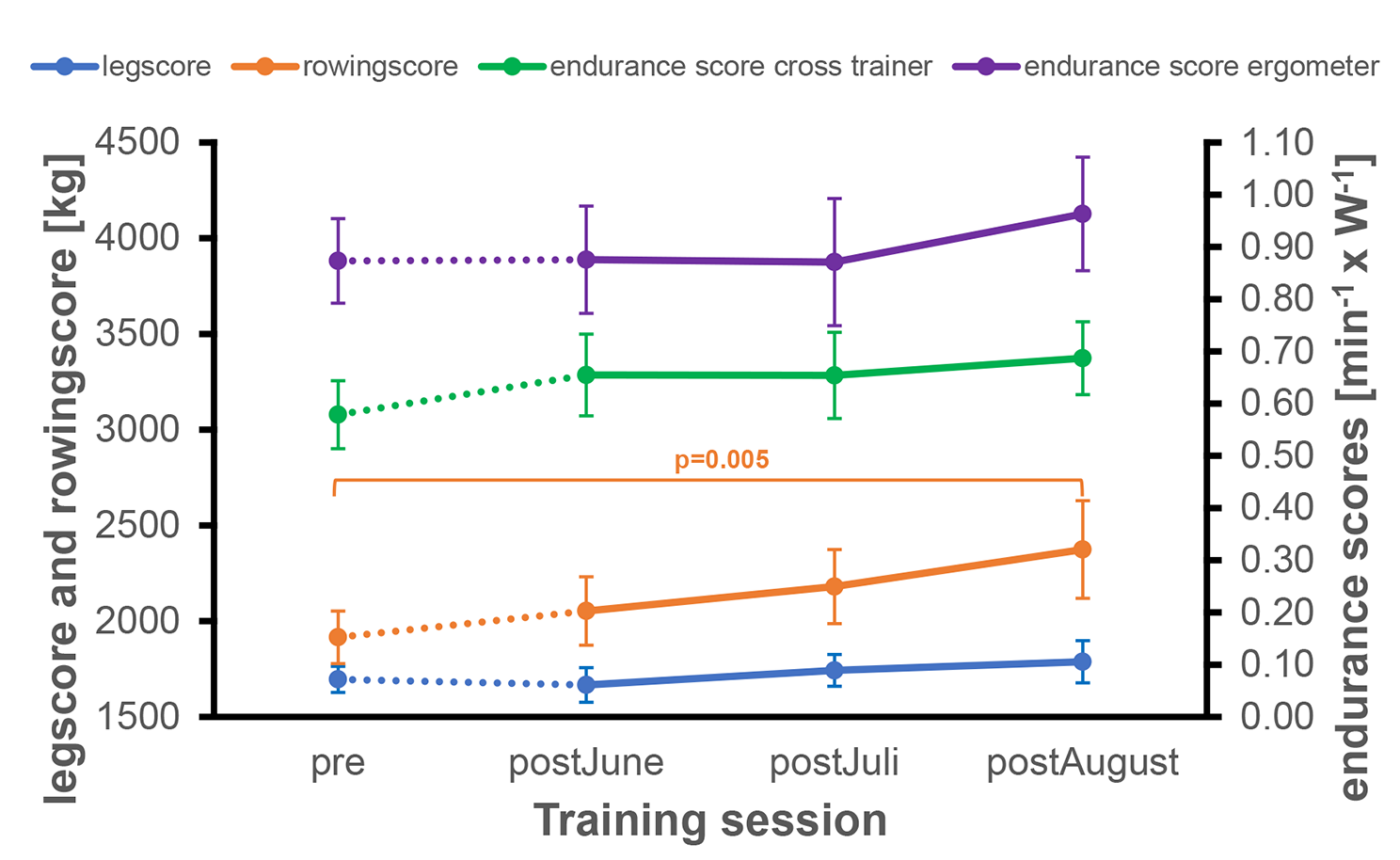
Leg score, rowing score and endurance scores of the subgroup *Time periods: pre: February to March 2020; post*_*June*_: *June 2020; post*_*July*_: *July 2020; post*_*August*_: *August 2020*.

In the subgroup analysis (n = 13), no significant differences were seen for the leg score and both endurance scores. For the rowing score, there were significant differences overall (p = 0,001), with significant differences between pre and post_August_(Fig. 5).

**Table 3:** Average (HR_mean_) and maximal (HR_max_) heart rates for the individual training devices

		mean values in absolute numbers [min^− 1^]					mean values in percent of the maximal heart rate			
**device**	**parameter**	**pre**	**post**_**June**_	**post**_**July**_	**post**_**August**_	**target HR**	**pre**	**post**_**June**_	**post**_**July**_	**post**_**August**_
**chest press**	HR_max_	101	99	99	101	149	68%	67%	67%	68%
	HR_mean_	96	95	94	96	149	64%	64%	63%	64%
**leg curl**	HR_max_	91	87	88	89	149	61%	58%	59%	60%
	HR_mean_	84	83	84	85	149	56%	55%	56%	57%
**leg extension**	HR_max_	101	102	100	100	149	68%	68%	67%	67%
	HR_mean_	95	96	94	94	149	64%	65%	63%	63%
**rowing**	HR_max_	110	110	109	107	149	74%	74%	73%	72%
	HR_mean_	105	105	103	102	149	70%	71%	69%	68%
**cross** **trainer**	HR_max_	122	121	118	120	149	82%	81%	79%	80%
	HR_mean_	111	110	108	109	149	75%	74%	73%	73%
**bicycle ergometer**	HR_max_	114	114	115	116	149	77%	77%	77%	78%
	HR_mean_	106	105	105	106	149	71%	71%	70%	71%
**overall**							69%	69%	68%	69%

*HR*_*max*_: *Maximum heart rate on the device; HR*_*mean*_: *mean heart rate on the device; target HR: maximum heart rate to achieve calculated by 220-age of the participant.*

*Time periods: pre: February to March 2020; post*_*June*_: *June 2020; post*_*July*_: *July 2020; post*_*August*_: *August 2020. 22–31 participants wore the belt to measure heart rate, depending on the device.*

### Correlation analyses

The explorative correlation analyses of total energy expenditure during physical activities and the individual leisure activities, revealed a weak correlation for walking (r = 0.341, p = 0.045), a moderate correlation for mowing the lawn (r = 0.411, p = 0.014), and cycling outside (r = 0.485, p = 0.003), and a strong correlation for gardening (r = 0.748, p ≤ 0.001) before the lockdown. During the lockdown, walking was weakly correlated (r = 0.337, p = 0.048), and gardening (r = 0.834, p ≤ 0.001) and cycling outside (r = 0.663, p ≤ 0.001) were strongly correlated with total energy expenditure. After the lockdown, significant correlations were seen with a strong correlation for gardening (r = 0.800, p ≤ 0.001), a moderate correlation for cycling outside (r = 0.584, p ≤ 0.001), and a weak correlation for cycling on the ergometer (r=-0.387, p = 0.022).

## Discussion

In this study, physical activities and health characteristics of a small sample of older individuals in the rural area of Oldenburg before, during and after the pandemic during the first lockdown period of the COVID-19 pandemic in Germany are described in combination with objective training data from a chip-controlled fitness circuit.

In contrast to the initial hypothesis, the data of this small sample show that the gym lockdown in Germany did not have a negative impact on the participants’ strength and endurance capacities on all exercise-training machines, and, in addition, an increase in outdoor physical activities could be seen. Rather, a slight training effect can be seen in the analyses despite the lockdown (Figs. 3a-d and 4a-b). For the resistance exercise machines, significant differences from February/March to July can be seen, especially on the seated rowing device, with an increase in performance (Fig. 3, b). Thus, with resumption of training, there was an increase in performance over time.

This is also observed for the cycle ergometer and cross trainer (Fig. 4, a-b) which is in contrast to previous studies, reporting a reduction in performance directly after the lockdown for endurance training [10,12].

The participants reached only ~ 67–68% of their age-adjusted maximal heart rate (Table 3). This heart rate range is recommended for maintaining individual fitness [23]. In order to increase their endurance performance, training should be performed at the anaerobic threshold within a heart rate range of 80–90% of maximal age-adjusted heart rate. However, as medication was not assessed in the questionnaires, we cannot control for heart rate lowering drugs. However pre-existing cardiovascular diseases were reported by 21 of 35 participants, hence rate control drugs, for example beta-blockers as one of the most prescribed drugs [20], are possibly taken by the participants. Despite the descriptive tendency and in some cases significant increase in performance, there was no significant reduction in heart rate on any of the machines. This is an indication against a strong increase in performance but could be an expression of the aim to preserve physical fitness.

For the analysed group of older adults, an average initial leg score of 1207 kg was documented. Hence, according to the results of Zieschang et al. [12], this group should be considered as moderately exercising individuals. There were no significant changes in the leg or the endurance scores, but performance on the rowing device increased over the whole period from preLD to postLD significantly without a drop directly after the lockdown.

In contrast, to the results of Zieschang et al., showing significant losses in upper and lower body exercise performance [12], the subgroup with intense training habits before the lockdown in this analysis did not show losses in exercise performance on the same training devices. We would have expected a significant drop in training performance during the lockdown. However, when comparing the mean values of the leg score of both studies, it can be seen that in the small subgroup of 13 people the leg score was 1696 kg and in the large study by Zieschang et al. it was 1892 ± 21 kg. The small subgroup of 13 people therefore had a lower baseline training performance, which may explain the different results. Another explanation could be that the participants spent more time working in the garden during the lockdown, which compensated for the absence of resistance training.

This is supported by the increasing energy expenditure over the three time points preLD over LD to postLD. This may be due to good weather conditions during the lockdown starting in spring. A more detailed analysis shows that the activities of walking, cycling and gardening in particular contribute significantly to the increase in energy expenditure and correlate strongly with overall energy expenditure (Table 2).The small sample analysed in this study, shows contrasting time courses of physical activity when compared with previously published studies that have clearly shown a decrease in physical activity during the lockdown period [1–3, 10,12]. This may be explained by the more rural character of Oldenburg with faster access to nature, larger gardens, a large network of hiking and biking trails and thus overall easier opportunities for outdoor physical activities than in a big city. However, there is no information about the living conditions in the other studies analysing the changes in physical activity during the Covid-19 pandemic [2–4, 10,12]. In addition to that, different restrictions during the lockdown in the other countries result in different possibilities for organising everyday life. From our data it is evident that the majority lives in single-family houses (85.7%). Hence, it can be concluded that the majority of the participants had access to a garden with associated work, which might be a difference to people living in urban areas.

The evaluation of the questionnaires revealed a significant decrease in the robust scores, as part of the LUCAS functional index (Fig. 2), and a decreasing trend in the psychological sum scale, calculated using the SF-12. Both are markers of social restraint [16,18]. The contact restrictions may have led to a subjectively reduced quality of life in the social domain. Subjectively, most of the participants were affected by the restriction of social contacts and not on financial burden or fear of an infection. This highlights the importance of social backup, especially for older people. In larger studies, analysing quality of life, it was shown that anxiety rises with physical inactivity during and after the lockdown, and led to higher stress [1–3]. Increased activity improves emotional, psychological and social well-being, contrary to our observations. In our study, it would have been expected that subjective well-being did not change with increased physical activity. One explanation could be that the increased physical activity of our subjects during the lockdown buffered at least part of the negative influence. It can be assumed that the negative influence of the restrictions during the lockdown on subjective well-being would have been even greater.

In contrast to previous studies, the participants interviewed in the Oldenburg area managed to maintain their exercise status and tended to improve it over the course of the observation period (February to August 2020). In particular, the three activities mentioned above, walking, cycling and gardening as the most improved activities, seem to be compensated the missing access to the fitness circuit. In the study by Zieschang et al., a significant decrease in lower and upper body strength had been shown [12] and several studies could show that strength training in particular has a greater protective effect on the musculature [14]. A possible explanation in our group here seems to be gardening as a correlate of strength training and outdoor cycling and walking activities for the endurance training. Gardening increased during the lockdown by 161 kcal/week, walking by 88.5 kcal/week and cycling by 181.5 kcal/week, in total 431 kcal/ week. This could balance the total calories of 265,5 kcal/week by the chip-controlled fitness circuit.

### Strengths and limitations

The strength of this study is the detailed description of physical activities assessed via questionnaires, combined with objective training data from endurance and resistive exercise devices. This allows a very detailed analysis of potential compensation strategies for the inaccessibility of structured sports activities. The interviews began closely, approximately six weeks after the lockdown and thus the retrospective recording of the individual situation before the lockdown appears to be acceptable.

Based on the demographic data (Table 1 and below), it can be seen that the 35 participants include all social groups of the society. However, this is a group that is generally voluntarily interested in maintaining its physical fitness and needs little support during daily life overall. In the study population 5.7% needed external care, while in the total population 7.6% are in need of a certain degree of external care in this age group [13].

The observation period of the study covers the end of winter to midsummer over three months. Thus, the results may be influenced by per se greater energy expenditure during the summer months and the results could change with longer lockdowns, especially over the winter months. This bias should be considered for the interpretation of the results. The calculated target heart rate, based on participants age, might not be reached due to drug use. This needs to be considered in future studies.

The study includes a small number of 35 participants, as direct recruitment of subjects by the study team was not possible. General statements on the overall population can therefore only be made to a very limited extent and the results should be treated with caution.

In addition, there is the possibility of a bias by participation in the study and the knowledge of the repeated interview about individual training habits. Participants might have paid more attention to their physical activity habits during the lockdown period. Furthermore, we have no information about the individual training intensity, whether the training was pushing the limits or not. Since the participants participated voluntarily in a fitness circuit and chose to participate in this study, the study population may be a selective sample of rather health-oriented older adults.

The main focus of this study was on the data from the chip-controlled fitness circuit. The creation of a control group with continuously exercising test persons was not possible due to the restrictions.

## Conclusions

Although our study group is small, we can objectively evaluate in detail the training course over several months before, shortly after and in the period after the onset of the pandemic using the training data from the chip-controlled exercise devices, and compare them with subjective and objective data of physical activity and well-being using the interviews. The results of the study showed that activities of daily living and outdoor physical activities during the lockdown conditions in Germany promoted the preservation of individual physical fitness.

Moderately active older adults living in a rural area can compensate for the lack of training through everyday activities such as gardening, walking and cycling. Therefore, this should be enforced or recommended in times of increased infection rates and the associated avoidance of fitness studios to maintain individual physical fitness levels.

## Data Availability

The datasets generated and analysed during the current study are not publicly available due to data protection restrictions, but are available from the corresponding author on reasonable request at geriatrie@uol.de.

## List of Abbreviations

pre: Time period containing milon data February to March 2020
post_June_: Time period containing milon data June 2020
post_July_: Time period containing milon data July 2020
post_August_: Time period containing milon data August 2020
preLD: Time period containing interview data before the lockdown
LD: Time period containing interview data during the lockdown
postLD: Time period containing interview data after the lockdown
Short FES-I: short Falls Efficacy Scale International
SF-12/SF-36: health status questionnaire containing 12 or 36 questions
LUCAS: Longitudinal Urban Cohort Ageing Study
sets: number of training sets
avg weight: average weight of the concentric load, while lifting the weight to the final position
avg delta weight: average weight of the eccentric load while releasing the weight back to the starting position
worksum: average work on the device
moves: overall number of repetitions on the device
avg moves: average number of repetitions for all sets of the session on the device
HR_max_: the maximum heart rate
HR_mean_: the average heart rate
duration: time spent on the device
avg rpm: revolutions per minute
avg watt: average power
BMI: body mass index (kg/m²)
kcal: kilocalories
